# The Importance of Early Diagnosis and Treatment to Limit the Impact of Dystrophic Cardiomyopathy—We’ve Seen This Film Before, and We Didn’t Like the Ending

**DOI:** 10.3390/jcdd12110416

**Published:** 2025-10-22

**Authors:** DeWayne Townsend

**Affiliations:** Department of Integrative Biology and Physiology, Medical School, University of Minnesota, Minneapolis, MN 55455, USA; town0045@umn.edu

**Keywords:** Duchenne muscular dystrophy, dystrophic cardiomyopathy, heart disease, prophylactic treatment

## Abstract

Duchenne muscular dystrophy (DMD) is a rare neuromuscular disorder that is characterized by skeletal muscle wasting, loss of ambulation, and respiratory failure. In addition to these obvious external signs of disease, heart disease, the leading cause of death in DMD patients, is also progressing. Dystrophic cardiomyopathy is largely clinically silent with cardiac dysfunction masked by concurrent loss of skeletal muscle function. In older DMD patients the prevalence of heart disease is very high, offering the unique potential to predict impending heart disease from a much earlier genetic diagnosis. Randomized clinical trial data and subsequent retrospective studies in DMD demonstrate that early initiation of cardiac directed therapy results in a significant delay in the onset of cardiac dysfunction and prolonged survival. Clinical guidelines reflect this, recommending the initiation of cardiac therapy at an age of 10 years old, even in the absence of documented contractile dysfunction. Despite this data, a recent examination of registry data reveals that most DMD patients are not receiving the treatments recommended by these guidelines. While there is great excitement regarding newly developed therapies for DMD, there are so many signs that deploying the safe and effective therapies we already have can improve clinical outcomes. This review will highlight the basic science behind and clinical importance of using early cardiac directed therapy to extend the duration and quality of life of DMD patients and will offer some suggestions that may aid in achieving this goal.

## 1. Introduction

There are relatively few medical conditions for which the diagnosis of disease precedes the onset of symptoms. However, with the advancement of widespread genetic testing, the potential to make diagnoses before any symptoms are evident is likely to grow. Given that most genetic diseases have no effective therapies, there are real bioethical debates about the value of pre-symptom testing. However, for a growing number of diseases, treatment options are available, and for these diseases, early treatment may have significant impact on disease progression, improving the quality and duration of patient lives. Duchenne muscular dystrophy (DMD) is one such disease.

DMD was first described in 1867 as a skeletal muscle disease with respiratory complications [1], but within a few years the involvement of the heart was also documented [2]. However, for well over a century the care and management of DMD was dominated by skeletal muscle and respiratory symptoms. During this time respiratory failure was the leading cause of death, and while heart disease was nearly ubiquitous, it was not treated as a life-threatening component of the disease. This began to change in the second half of the 20th century as the management of respiratory disease improved and life expectancies of DMD patients continually increased [3,4]. At the turn of the century, it became apparent that heart disease was becoming the leading cause of mortality in DMD patients.

## 2. Pathophysiology of Dystrophic Cardiomyopathy

One hundred and twenty years after its first description, the gene responsible for DMD was identified [5,6]. The resulting protein, named dystrophin, was found to be central for the organization of a complex of glycoproteins in the surface membrane of striated muscle cells [7,8,9,10]. Together, dystrophin and its associated glycoprotein complex function as a bridge between the internal cytoskeleton and the extracellular matrix [11,12]. While the details of all of dystrophin’s functions remain incompletely understood, its role in preserving the membrane integrity of striated muscle cells is well documented [13,14]. This aspect of dystrophin’s function was supported by both its biochemical associations spanning the muscle membrane and by the clear evidence of muscle injury present in the muscles of DMD patients.

### 2.1. Muscle Injury—Skeletal Muscle vs. Myocardium

From an evolutionary perspective, any given skeletal muscle cell is relatively dispensable. Most of them are found on limbs that are readily exposed to the external environment and potential trauma. As such, elaborate mechanisms have evolved to replace injured skeletal muscle, highlighted by satellite cell differentiation into mature muscle fibers [15]. As anyone who has experienced muscle injury will know, during this repair process force development by the injured muscle is greatly reduced. In contrast, the cardiomyocytes of the heart are relatively protected inside of a boney cage and surrounded by air filled lungs. As such the potential for non-fatal traumatic injury of the heart is relatively low. Accordingly, the myocardium does not need to be very good at regeneration. This is evident in the case of myocardial ischemia where blood flow to portions of the heart is temporarily disrupted, resulting in permanent loss of cardiomyocytes [16]. Even in the dystrophic heart, where blood supply is not interrupted, evidence of cardiac regeneration is very scarce. Instead, the heart’s primary response to injury appears to be to rapidly replace injured cells with fibrosis [17,18,19]. The rapid placement of load-bearing extracellular matrix appears to ensure that force transmission across the injured myocardium is maintained, thus maintaining contractile force throughout the heart and preserving cardiac output despite the injury. It is possible that this robust fibrotic response may create an environment unfavorable to myocyte regeneration, as aggressive fibroblast response to cardiac injury is coincident with the loss of cardiac regeneration [20]. In summary, the poor regenerative capacity of the heart following injury highlights the irreversible nature of cardiac injury and the importance of preventing myocyte loss as a means of limiting progressive cardiomyopathies, such as that seen in DMD.

### 2.2. Myocardial Dysfunction and Mortality

For over a century the most common cause of death in DMD patients was related to respiratory failure [4]. This is the result of both reduced ventilation, due to the severe pathology present in the diaphragm, and the inability to cough, driven by widespread weakness in the abdominal and intercostal musculature. Through advancements in technology and improvements in clinical management of airways in individuals with DMD, life expectancy has greatly improved. It is notable that recent data indicates that overall survival is largely uncoupled from respiratory function but is more closely linked to cardiac capabilities [21,22,23]. This data highlights the importance of persevering cardiac function in the context of DMD. Given that cardiac dysfunction in DMD is derived from the progressive loss of functional myocardium and that this loss is essentially irreversible, it strongly suggests that prevention of cardiac disease will lead to further increases in life expectancy. To understand that challenges in preventing cardiac injury in DMD, we must understand when dysfunction starts.

## 3. Diagnosis and the Onset of Dystrophic Cardiomyopathy

The diagnosis of DMD typically occurs by the age of 5 years but varies between countries [24,25,26]. The reasons for delays in diagnosis are multifactor, including socioeconomic status and healthcare availability [26,27]. The importance of early diagnosis is becoming evident as the effectiveness of early treatment with glucocorticoids appears to provide additional benefit [28,29]; however, additional studies are needed to solidify the long-term impact of early steroid treatment. The implementation of specific dystrophin replacement strategies that may be more feasible in younger DMD patients would also be facilitated by earlier diagnosis [30]. In fact, there are significant ongoing efforts to implement newborn screening for DMD in the US, already implemented in Minnesota and Ohio and pending across the United States [31] and the world [32,33], with the aim of lowering the age of initial diagnosis.

Defining the earliest stages of cardiomyopathy in DMD is challenging, as there are many confounding variables and distinct phenotypes that may be present. The prevalence of cardiac dysfunction in older DMD patients is essentially universal [34], usually defined by reductions in ejection fraction. Ejection fraction is relatively easy to obtain by echocardiography but only becomes abnormal after disease has significantly advanced. Echocardiographic assessment of diastolic function demonstrates that DMD patients demonstrate diastolic dysfunction very early in the course of the disease [35]. The importance of diastolic dysfunction as an early marker of disease is also evident in cardiac magnetic resonance (CMR) imaging [36]. The use of CMR imaging in the evaluation of dystrophic cardiomyopathy is growing; however, there are significant limitations to its use, including higher cost and lower availability relative to echocardiography. Furthermore, the prolonged time required to obtain CMR imaging makes it challenging for young patients, who often would need to be sedated, and those with scoliosis and contracture for whom lying in the scanner can be painful. Despite these limitations CMR imaging can provide a measure of myocardial scar tissue using late gadolinium enhancement (LGE). Serial imaging studies reveal that the presence of LGE precedes global contractile dysfunction [37] and its magnitude increases as cardiac contractile function declines [38]. The resolution of a standard LGE scan is ≈18 mm^3^, a volume that encompasses over a million cardiomyocytes [39], indicating that even with optimal methodologies, significant injury scattered through the myocardium may go undetected. The complex architecture of the heart results in unique strains throughout the myocardium. Specifically, segmental circumferential strain (ε_cc_) in the left ventricle is an early marker of cardiac dysfunction evident in DMD patients as young as 6 years of age [40,41,42]. It is notable that the regions of the heart demonstrating the largest abnormalities in ε_cc_ are the same regions that later accumulate areas of LGE. The mechanisms underlying the reduced circumferential strain are unknown. It may result from the accumulation of small regions of cardiac fibrosis secondary to myocyte death that are too small to be detected by LGE yet together alter heart function. However, it is also possible that the reduced strain may result from attenuated force production by dystrophic cardiomyocytes or disruption of force transmission through the heart in the absence of dystrophin [43]. Regardless, these significant global manifestations of cardiac dysfunction are present in the youngest DMD patients evaluated [41], indicating that in many patients, the disease process is well established even before 10 years of age.

## 4. Treatment of Dystrophic Cardiomyopathy

The medical therapy of heart disease in DMD has developed into stage-dependent treatment approaches: (1) prior to the onset of any cardiac dysfunction, (2) following the identification of cardiac dysfunction, and (3) in response to symptomatic heart failure. The specifics of these therapies have been thoroughly reviewed elsewhere [44,45,46,47,48,49]. In short, early treatments rely on the most proven effective therapies for dystrophic cardiomyopathy that are directed at disrupting the renin-angiotensin system, with angiotensin converting enzyme inhibitors (ACE-I) having the most supporting clinical data. Angiotensin II receptor blockers (ARBs) also have been shown to slow the progression of heart failure in DMD [50]. Once disease begins to be evident, evidence supports a potential therapeutic benefit for aldosterone inhibitors [51] and β-adrenergic receptor antagonists [52]. Recently, there have been reports of the use of advanced cardiac therapies (e.g., ventricular assist devices) to treat advanced dystrophic cardiomyopathy [53]. The introduction of specific dystrophin-replacement therapies offers the promise of a permeant cure. Ongoing trials of these approaches have general cardiac functional end points, but the young age of the patient populations enrolled means that it will likely be decades before the cardiac efficacy of these therapies is known.

## 5. Prophylactic Treatment of Dystrophic Cardiomyopathy

### 5.1. Support for Prophylactic Treatment of Dystrophic Cardiomyopathy

The dysfunction of cardiac contraction in DMD is caused by the loss of functional myocardium and its replacement with scar tissue. The presence of this scar within the myocardium likely increases the strain on neighboring myocytes, increasing the rate of their death and the growth of fibrotic lesions in the dystrophic heart. Two pivotal reports of a single trial aiming to understand the effectiveness of proactive treatment of dystrophic cardiomyopathy have fundamentally changed how we treat this disease [54,55]. The trial began with 80 ≈10-year-old DMD patients. A quarter of them were excluded because their cardiac function was already impacted by disease progression. The remaining participants were randomly divided in a double-blind fashion; one group received an ACE-I for three years while the other group received a placebo. Following this three-year trial, period all participants were treated with ACE-I in an open label phase. After the first three years of the trial, the double-blind period, there were no significant differences in ejection fraction between the groups, with nearly all patients having ejection fractions >45%. This is notable, as under the previous recommendation, most of the patients in this trial would not have been recommended for therapy. However, after five years, with the participants being ≈15 years old, significantly more patients in the delayed treatment group developed cardiac dysfunction. By the time these participants were ≈20 years old, those that started ACE-I at 10 years of age were significantly protected from death. This data is further supported by registry data demonstrating that DMD patients receiving preventative ACE-I therapy were protected from the development of heart failure and death [56].

Subsequent studies have explored the impact of additional therapeutics the prophylactic actions of ACE-I. A recent set of studies mirrored the original prophylactic study, enrolling participants with DMD at ≈10 years of age; however, in addition to an ACE-I, a β-blocker was also provided. Like the initial study, this combined therapy was well tolerated and showed no evidence of protection at 36 months [57]. In the original prophylactic study, evidence of cardioprotection was present by 60 months; however, the combined β-blocker/ACE-I trial failed to observe a similar beneficial effect at the 5-year time point [58]. While trends leaning toward a therapeutic benefit are present, the preservation of cardiac function in the delayed treatment group of the recent study is the main driver. The authors suggest that increased use of steroids in the delayed treatment group may be preserving cardiac function to a greater extent than the original study [59,60]. It is notable that the original prophylactic cohorts continued to separate, with significant mortality benefit becoming evident 10 years following the initiation of the study; thus, extended follow-up times may be required to observe differences as standard of care prolongs the periods of normal cardiac functions in DMD patients. An additional study of combined ACE-I and β-blockers in older patients demonstrated a small improvement in ejection fraction in the combined therapy group, while the ACE-I alone maintained cardiac function during the three-year follow-up period [61].

Another study was conducted in boys with DMD that had intact cardiac function, but evidence of myocardial injury—detected by LGE. These inclusion criteria resulted in an older participant population of ≈15 years of age. This study evaluated the effectiveness of the mineralocorticoid receptor antagonist (MRA) eplerenone to improve cardiac function on top of a background of ACE-I/ARB treatment. After a year of treatment, the eplerenone groups had small, but statistically significant improvements in ε_cc_ and ventricular ejection fraction [51]. An open-label extension trial with a subset of the original cohort was performed. By random chance, the participants carrying over into the second study, those that received the placebo (in addition to ACE-I/ARB therapy) in the initial study, were significantly younger than the participants receiving eplerenone. This allowed two questions to be addressed. (1) What is the impact of the addition of eplerenone treatment to young patients with evidence of cardiac injury? (2) What are the effects of prolonged eplerenone treatment in older participants with DMD? The results of this study indicate that ε_cc_ is significantly improved in the first year of eplerenone treatment, even if that treatment is delayed. This improvement was maintained during the second year of follow-up, but the effect appears to plateau. A similar plateau effect was observed in the older participants that had been receiving eplerenone during the initial trial where ε_cc_ is relatively stable during the two years of follow up [62]. The results of these studies suggest that MRAs provide a benefit to patients with existing cardiac injury, and given aldosterone’s pro-fibrotic actions, this makes sense. It is less clear if MRAs would benefit DMD patients prior to the onset of LGE-documented cardiac injury. Finally, the relatively short time frame of these studies limits our understanding of the long-term effects of prolonged MRA on dystrophic cardiomyopathy. While efficacy remains in question, these studies and others [63] clearly demonstrate the safety of adding MRAs to existing therapies.

The demonstrated benefit of prophylactic treatment of dystrophic heart disease has led to changes in the recommendations for the management of heart disease in DMD patients [44,45]. These current guidelines include annual monitoring using non-invasive imaging modalities. The preferred modality is CMR, but echocardiography is also acceptable, especially in younger patients for which the long acquisition times and breathing protocols of CMR can be challenging. Mirroring the design of the trial discussed above, an NHLBI Working Group recommended beginning treatment with ACE-I at 10 years of age, even if evidence of cardiac dysfunction is not detected, although there is some debate regarding if the risks of chronic ACE-I treatment outweigh the potential benefits of preventing the onset of a disease process that may not have started [45].

### 5.2. Challenges with Initiating Prophylactic Treatment for Dystrophic Cardiomyopathy

A recent analysis of clinical data from Muscular Dystrophy Association-sponsored clinics reveals that prophylactic treatment of heart disease in DMD patients has become more common since the publication of guidelines recommending early treatment; however, the majority of DMD patients are not receiving prophylactic treatment [64]. The decision to begin treatment is further complicated by the highly variable onset of cardiac dysfunction [65,66] that is difficult to predict [21,66]. Another important concern is that the most common diagnostic tool, the echocardiogram, is relatively insensitive in the detection of early-stage cardiomyopathy. While CMR has the capabilities to detect scar formation, via LGE, and early contractile dysfunction, via circumferential strain, it is not as readily available. Another concern is that ACE-I has significant adverse effects. In adults, ARBs have fewer side effects [50] and thus may be a suitable substitute; however, it is not clear if this will be the case in a pediatric population, and while ARBs are effective in slowing disease [67], they have not yet been shown to be an effective prophylactic therapy. Finally, the potential for renal disease is another significant concern that could be complicated by prolonged ACE-I or ARB treatment [68,69].

## 6. Pathophysiology of Delayed Treatment in Dystrophic Cardiomyopathy

**Figure 1:**
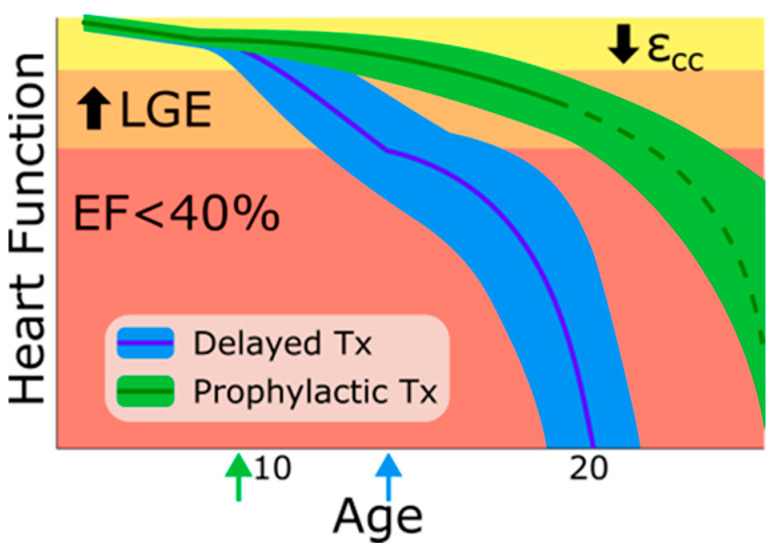
Schematic of the impact of the timing of therapy initiation on the development of cardiac dysfunction in DMD. Based on clinical data the earliest sign of cardiac dysfunction is a reduction in circumferential strain (ε_cc_) followed by accumulation of late gadolinium enhancement (LGE) as cardiac fibrosis continues to expand. Global contractile dysfunction, ejection fraction (EF) < 40%, is only evident after years of accumulated injury. The prior practice of waiting until global cardiac dysfunction is present (blue arrow) results in poorer cardiovascular outcomes. Randomized clinical studies suggest that even a delay of three years has significant impact on cardiac function and all-cause mortality outcomes in DMD patients; in contrast, prophylactic cardiac directed therapy (green arrow) results in delays in onset of cardiac dysfunction and improved survival. See the main text for details. The extent of the protection provided by early treatment is currently unknown (dashed line) but likely will not be able to fully reverse the disease process.

Heart disease is nearly universal in DMD patients [34,65,70] and under modern treatment regimes, the function of the heart is closely correlated to overall survival [22,66,71]. Thus, it appears that preserving cardiac function is critical for continuing the trend of expanding the life expectancy of DMD patients. As outlined above, the progressive nature of dystrophic cardiomyopathy is driven by the irreversible loss of viable myocardium and the accumulation of cardiac fibrosis. This model of progression is highly compatible with the clinical presentation of heart disease in DMD patients. Initially, when the areas of damage are few and scattered, cardiac function is largely preserved, with only modest changes in strain measurements within the heart. Over time more myocardium is lost, often in areas neighboring existing lesions, causing fibrosis to expand. Soon these areas are sufficiently large to be detected via LGE. These areas of cardiac fibrosis continue to accumulate and eventually reach a threshold where the cardiac reserve is completely spent and defects in contractile function are observed (Figure 1).

The conundrum is that we must begin treating the heart disease of DMD before it is having an impact on global cardiac function. Being a rare disease there is a dearth of prospective clinical trial data for DMD; thus, the decision of when to initiate cardiac therapy must be made with incomplete information. Clinical guidelines have been issued and have impacted the care of many DMD patients, with earlier screenings and earlier medical treatment in a third of patients in the US [64]. However, this leaves two-thirds of DMD patients at MDA-sponsored clinics not receiving care, in accordance with prior guidelines. Importantly, this is occurring at clinics specializing in neuromuscular diseases, raising concerns that rates of guideline-directed therapy of heart disease in DMD might be even lower at non-specialty clinics.

## 7. Next Steps

There is little data regarding the factors leading to the withholding of prophylactic cardiac directed treatment, but reluctance to prescribe drugs to treat a condition that has not yet manifested is likely a large contributor. More aggressive utilization of CMR may provide additional information for providers reluctant to treat based solely on a genetic diagnosis. Another potential cause of deviation from the guidelines could be a lack of knowledge. This may be especially important for providers outside of specific muscular dystrophy clinics who may only see a few DMD patients in their entire career. It is possible that patient advocacy groups may have an impact through providing additional educational information, for both providers and families, regarding the data supporting early treatment of dystrophic cardiomyopathy. Bringing more information to the conversation about when to start prophylactic cardiac therapy will allow a more informed assessment of the pros and cons of this important clinical decision.

## 8. Conclusions

In contrast to most of cardiology, with dystrophic cardiomyopathy it is not a question of if, but rather of when heart disease will present itself. The moment a genetic diagnosis of DMD is obtained, it is almost certain that cardiac dysfunction will manifest in the next dozen or so years. Randomized clinical trial data highlights the effectiveness of early therapy for slowing the onset of cardiac dysfunction. However, the most effective timing to initiate therapies is not clearly known, primarily because a wide variety of factors contribute to significant variability in the progression of dystrophic cardiomyopathy. There is currently much excitement regarding the promise of dystrophin replacement therapies; however, the trials evaluating the effectiveness of these are not designed to directly assess cardiac function and it will likely be many years before we really know the answers [72]. Furthermore, there are significant concerns that these replacement strategies will be ineffective in the heart, on top of the significant safety concerns regarding therapy delivery. For DMD patients, prophylactic therapy may provide them a safe and effective approach to change the course of their heart disease and extend their lives.

## Data Availability

Not applicable.
